# Rethinking of phosphodiesterase 5 inhibition: the old, the new and the perspective in human health

**DOI:** 10.3389/fendo.2024.1461642

**Published:** 2024-09-17

**Authors:** Maria Paola Paronetto, Clara Crescioli

**Affiliations:** ^1^ Department of Movement, Human and Health Sciences, University of Rome Foro Italico, Rome, Italy; ^2^ Laboratory of Molecular and Cellular Neurobiology, Fondazione Santa Lucia, IRCCS, Rome, Italy

**Keywords:** PDE5i, cGMP, inflammation, cardiac diseases, cancer

## Abstract

The phosphodiesterases type 5 (PDE5) are catalytic enzymes converting the second messenger cyclic guanosine monophosphate (cGMP) to 5’ GMP. While intracellular cGMP reduction is associated with several detrimental effects, cGMP stabilization associates with numerous benefits. The PDE5 specific inhibitors, PDE5i, found their explosive fortune as first-line treatment for erectile dysfunction (ED), due to their powerful vasoactive properties. The favorable effect for ED emerged as side-effect when PDE5i were originally proposed for coronary artery disease (CAD). From that point on, the use of PDE5i captured the attention of researchers, clinicians, and companies. Indeed, PDE5-induced intracellular cGMP stabilization offers a range of therapeutic opportunities associated not only with vasoactive effects, but also with immune regulatory/anti-inflammatory actions. Chronic inflammation is acknowledged as the common link underlying most non-communicable diseases, including metabolic and cardiac diseases, autoimmune and neurodegenerative disorders, cancer. In this scenario, the clinical exploitation of PDE5i is undeniably beyond ED, representing a potential therapeutic tool in several human diseases. This review aims to overview the biological actions exerted by PDE5i, focusing on their ability as modulators of inflammation-related human diseases, with particular attention to inflammatory-related disorders, like cardiac diseases and cancer.

## 1 Introduction

The enzyme family of phosphodiesterases (PDEs) catalyzes the hydrolytic breakdown of cyclic adenosine monophosphate (cAMP) and cyclic guanosine monophosphate (cGMP) into the biologically inactive derivates 5′-AMP and 5′-GMP, respectively. In Eukaryotes, through their activity instrumental to regulate cAMP/cGMP intracellular levels, PDE5 can control the homeostasis of a variety of intracellular signaling and, therefore, multiple cellular functions.

Mammalian PDEs comprise 11 subfamilies, encoding distinct PDE isoforms through alternative pre-mRNA splicing, multiple promoter usage and alternative transcription start sites in human, rat, and mouse (1).

In human tissues, PDEs are almost ubiquitous and share the main catalytic function to hydrolyze the 3′ cyclic phosphate bond of cAMP, cGMP, or both, but with different substrate specificities and intracellular localization (2, 3).

PDE enzymes can be grouped into three broad categories based on their sequence homology, substrate specificity and selectivity: PDE4, PDE7 and PDE8 specific for cAMP hydrolysis; PDE5, PDE6 and PDE9 specific for cGMP hydrolysis; PDE1, PDE2, PDE3, PDE10 and PDE11 exhibiting dual specificity, for cAMP and cGMP, although with different affinities, depending on the isoform.

PDEs are structurally modular proteins sharing a conserved catalytic core of approximately 270 amino acids. The determinants responsible for subcellular localization and specific interactions of the isoforms are in the N-terminal portions; these regulatory regions can be post-translationally modified and modulate the enzymatic activity/localization in response to external and internal cues. In addition, the N-terminal domain can include dimerization domains and autoinhibitory modules, as in PDEs 1, 4, and 5.

PDEs function is not limited to cyclic nucleotide content regulation within cells, it rather allows the single cell to respond to and interact with intra- and extracellular signals, through the creation of subcellular compartmentalization (like individual pockets, nanodomains) of cyclic nucleotide signaling (1). Indeed, PDE location might change depending on several factors, i.e., tissue type, health/disease status or aging; this characteristic is of paramount importance when considering PDEs as potential therapeutic targets (1, 4–10). In this view, many companies exploited the ability of PDEs to control specific paths to develop selective drugs against specific PDE types and isoforms (11). The efficacy of the prototypical “first-in class” PDE inhibitors (PDEi) emerged in the early ‘70s, the first-generation of PDE inhibitors was produced between the 80s and 90s, followed by the development of highly selective isoenzyme-specific drugs (11). Indeed, since PDE dysfunction associates with several pathophysiological conditions affecting, i.e., cardiovascular system, fertility, nervous system, metabolism immunity and cancer, PDEs represent potential therapeutic targets in several human diseases (1).

Among the different inhibitors, PDE5i undeniably represent the first and most successfully studied molecules (1). Originally proposed for the treatment of CVD, these drugs found their explosive fortune for erectile dysfunction (ED) treatment, due to their vasoactive activity. Nowadays, PDE5i are licensed for ED and pulmonary artery hypertension (PAH). Importantly, PDE5i can efficaciously counteract inflammatory processes and can regulate cell growth/division/death (12). Based on these properties, the clinical exploitation of PDE5i might be undeniably beyond ED, with important implications in several human diseases. In fact, chronic inflammation, or meta-inflammation, is widely recognized as the common link in most non-communicable diseases (NCD).

This review aims to overview and highlight the biological actions exerted by PDE5i, focusing on their ability as modulators of inflammation-related human diseases, with particular attention dedicated to inflammatory-related disorders, cardiac diseases and cancer.

## 2 PDE5 and PDE5 inhibitors

PDE5 belongs to the metallo-hydrolase superfamily and controls different physiological function through cGMP to 5’ GMP catalytic conversion (13).

PDE5 dimeric structure includes the regulatory unit that expresses GAF-A and GAF-B domains, acting in concert to control the catalytic unit and PDE5 dimerization (14). The N-terminal GAF-A domain exhibits cGMP binding sites, which account for PDE5 affinity to cGMP (15). Many human tissues express PDE5, i.e., visceral and vascular smooth muscle, including corpora cavernosa, skeletal muscle, platelets, lung, brain, spinal cord, kidney, gastrointestinal tissue, prostate, bladder, urethra (14, 16–22).

The *PDE5A* gene, located on the human chromosome 4q26, contains 23 exons spanning approximately 100 kilobases and encoding three alternatively spliced coding variants (*PDE5A1,2,3*), which display different isoform-specific first exons, guided by specific promoters responsive to cGMP or cAMP stimulation (16, 23).

In mice, the splice variants of the PDE5A, *mPde5a1*, *mPde5a2*, and *mPde5a3* show differential tissue distribution; the adult heart express different levels *mPde5a1* and *mPde5a2*, while *mPde5a3* seems undetectable (24).

In humans, PDE5A3 protein expression is restricted to smooth and cardiac muscle, while PDE5A1 and PDE5A2 are ubiquitously expressed (16).

The three PDE5A isoforms share the same cGMP-catalytic activities and differ only in the N-terminal region. All isoforms are similarly inhibited by sildenafil, a specific PDE5i, marketed as a treatment for ED in 1998. In fact, due to its selective inhibition of cGMP degradation in vascular smooth muscle cells in corpora cavernosa, sildenafil promotes vascular dilatation through nitric oxide (NO) increased availability (25). Thereafter, between 2003 and 2012, the other specific PDE5i tadalafil, vardenafil and avanafil were licensed for ED treatment (26). Currently, several PDE5i are FDA approved in and outside United Stated and marketed for ED (1).

Since cGMP is a second messenger involved in many physiological processes, beyond vasodilation, it is not surprising that cGMP signaling modulation might represent a useful tool to control other pathophysiological conditions.

**Figure 1:**
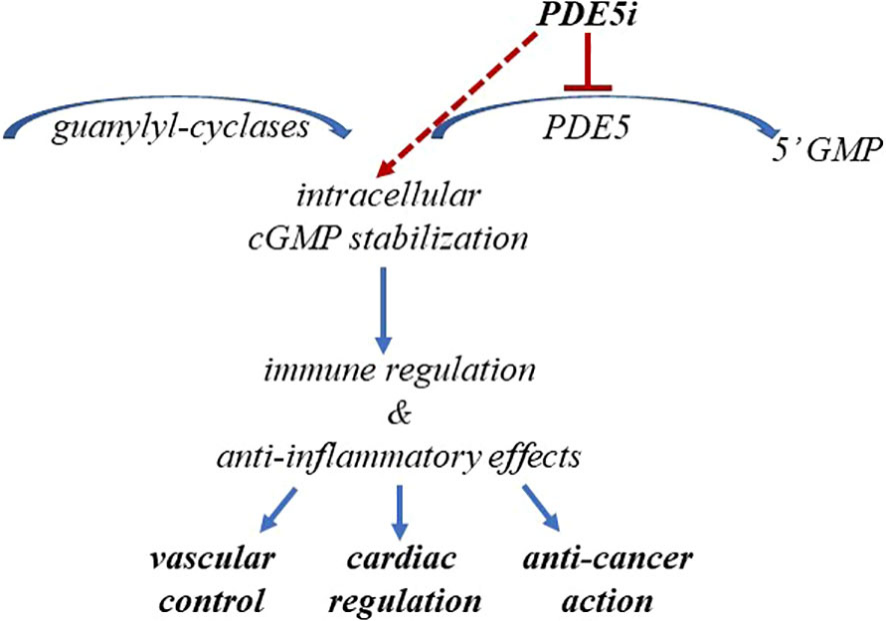
PDE5i-induced cGMP stabilization impacts human health. The figure schematically summarizes the benefits from cGMP stabilization in cardiovascular diseases and cancer.

Indeed, cGMP stabilization, beside benefits on endothelial, associates with several downstream favorable effects, as addressed later in this paper. Furthermore, the observation that cGMP accumulation can counteract inflammatory processes might be clinically relevant, considering that systemic chronic inflammation is the leading cause in several diseases, including neurological disorders, PAH, hypertension, cardiomyopathy, cancer, and lower urinary tract syndrome, in addition to ED (27–32) as summarized in **Figure 1**.

## 3 The role of PDE5i in inflammation: a roundtrip from cardiac disease and erectile dysfunction

Since quite long ago, anti-inflammatory drugs have been recognized as useful tools to control and counteract the risk of cardiovascular diseases (33). The homeostasis between cardiovascular health and disease largely depends on the immune/inflammatory balance associated with the specific biomolecular mediators, such as cytokines and chemokines. Inflammation triggers the early phases of the atherosclerotic processes and increases the level of inflammatory mediators, that, in turn, associates with a higher risk of developing cardiovascular diseases. Several contributions sustain the proof of concept that inflammation and especially inflammatory cytokines represent a significant risk factor of cardiovascular event development in different conditions, including primary heart diseases, myocarditis secondary to infections, metabolic diseases or rheumatic diseases (34–36). The description of the role of inflammatory cascades involved in distinct conditions in heart diseases goes beyond the aim of this review; we remand therefore to exhaustive reviews focusing on this topic (37–41). In this context, the need to target inflammation emerges as a therapeutic strategy to manage cardiovascular diseases.

Even if PDE5i represent a milestone in ED treatment, this class of drugs was originally developed in the late ‘80s for the relief of angina pectoris (42, 43). Sildenafil was indicated for the treatment of coronary artery disease (CAD), in 1989. While the beneficial effects were quite disappointing for CAD, interesting vascular effects on male penile erection emerged (44).

Furthermore, the observation that PDE5 inhibition can also ameliorate pulmonary vascular resistance (45) led to a secondary approval of sildenafil and tadalafil for PAH treatment (1, 46).

Albeit the vascular targeting was the main indication for therapeutic purposes in ED and PAH, PDE5i-induced regulatory properties led to “rethinking” of their application for cardiac purposes. Thus, albeit initially the concerns on the use PDE5i for CAD treatment - due to the marginal beneficial effects - shifted their application from CVD to ED, thereafter, sildenafil was “reallocated” for the treatment of cardiac disorders, due to both endothelial and anti-inflammatory/pro-remodeling effects in cardiac cells (43).

The clinical relevance of PDE5i has been proven in ischemia-reperfusion injury, pressure overload-induced hypertrophy, heart failure, myocardial ischemia (47, 48). Undeniably, PDE5i-induced benefits on cardiac health can be explained by endothelial function improvements exerted by these drugs throughout the body. Vascular endothelial cells and vascular smooth muscle cells are the canonical targets of NO–cGMP signaling, which, when potentiated by PDE5i, results in cGMP stabilization, and, consequently, in vasodilatory, antioxidative and anti‐proliferative effects (49).

The vasodilatory properties of the NO–cGMP pathway can reduce peripheral vascular resistance and improve cardiac preload to ameliorate myocardial perfusion, resulting in clinically relevant advantages, like in ischemic heart disease. PDE5 expression is found in cardiac vessels, i.e., coronary vascular smooth muscle cells (50, 51). However, the cardiac benefits from PDEi-induced cGMP signaling potentiation are reported in cardiomyocytes as well (52–54).

While PDE5 is almost undetectable in heathy cardiomyocytes, localized at Z-disks, enzyme expression is significantly upregulated and diffusely distributed in pathologic conditions, such as congestive heart failure, right ventricular hypertrophy, overload hypertrophy, ischemic cardiomyopathy (55–58). As from previous studies, isolated human cardiomyocytes express PDE5 similarly to human endothelial cells and human peripheral blood mononuclear cells (59).

Thus far, PDE5 is likely upregulated during cardiac remodeling processes leading to diseases. Cardiac dysfunctions, albeit distinct, associate with “aberrant” cardiac remodeling, that comprehends many pathophysiological changes at cell level, including, i.e., cell death, energy metabolism, oxidative stress, inflammation, collagen and contractile protein content, calcium transport (60). Growing evidence shows that inflammation and oxidative stress play a pivotal role in these processes. The immune/inflammatory response represents *per se* a physiological adaptation aimed to restore homeostasis after short-term stress, but, under persistent stressful stimuli - i.e., due to metabolic morbidities, pressure/volume overload, injury, or overtraining – the consequent cellular and molecular modification result in cardiac loss function, at first asymptomatic, ending in heart failure (61–64). Of interest, cGMP/cGMP-dependent protein kinases G (PKG) signaling, which is enhanced by NO-soluble guanylate cyclase (GC) and natriuretic peptide NP-particulate GC (pGC), regulates cardiac remodeling and limits inflammation (42, 65). I.e., heart failure either with reduced ejection fraction or preserved ejection fraction (HFrEF and HFpEF, respectively) is characterized by impaired and dysfunctional cGMP-PKG signaling (65).

Following PDE5i, cGMP concentration rises and activates PKG, which, in turn, deactivates various cellular signaling cascades engaged in aberrant remodeling and hypertrophy development, through suppression of effects mediated by calcineurin, protein kinase C (PKC), Ras homolog gene family member A (RhoA) and Rho-associated kinase (ROCK), and extracellular signal regulated kinase (ERK)-mitogen-activated protein kinase (MAPK) (65).

Remarkably, sildenafil either combined or not with natriuretic peptide (NP), that stimulates *per se* cGMP production, phosphorylates sarcomeric proteins, such as titin, troponin-I, cardiac myosin-binding protein C, reducing myocyte/myofiber stiffness and improving systolic and diastolic function (66–69). Hence, PDE5 inhibition emerges to play a pivotal role against cellular processes underlying disease initiation and development, by counteracting biomolecular cascade and mediators engaged in shift from health to disease. This effect, in part, relies on the reduction in T helper (Th)1-type mediators dependent on cGMP stabilization (70), as addressed in the following paragraph.

### 3.1 cGMP stabilization, immune/inflammation and impact on health

Since cGMP stabilization inhibits inflammatory processes, it represents a potential tool to limit initiation/progression of several diseases driven by inflammation, including metabolic and cardiovascular diseases, autoimmune and neurodegenerative disorders, and cancer (27–30). Indeed, in various cell types, cGMP accumulation dependent on PDE5 inhibition associates with several therapeutic benefits, as previously addressed (13). Thus, the use of PDE5i has been pharmacologically exploited beyond ED and PHA.

We briefly recall that cGMP synthesis occurs upon NO stimulation and, through GC, activates downstream cGMP-PKG and ion channels with a variety of effects (71).

The reduction of intracellular cGMP is well known to be involved in detrimental events in impaired vascular responses, vascular smooth muscle proliferation, in enhanced platelet aggregation and in immune response (72–75). In immunity, cGMP signaling pathway acts through the modulation of nuclear factor (NF)-κB and plays a role in critical processes, i.e., stress, inflammation, immune cell differentiation and regulation tolerance (76, 77). The experimental increase of cellular cGMP (through a NO donor) results in the reduction of inflammatory cytokines, like TNFα or IL-6, which, conversely, are increased when cGMP is reduced (by a soluble GC inhibitor) (78). Albeit some roles and mechanisms are still to be fully elucidated, it is documented that the reduction in cGMP formation suppresses cGMP-induced anti-inflammatory effects in experimental conditions mimicking inflammation (79).

Furthermore, sildenafil-induced cGMP increase can impact the regulation of T cells and the production of proinflammatory cytokines (80). Animal studies report that sildenafil decreases serum IL-6 and impacts T cell subpopulations - i.e., CD4+/CD8+ T cell ratio, T regulatory (Treg), natural killer (NK) T cells, with some sex-dependent effects (81). The anti-inflammatory effect was confirmed by murine studies on neuroinflammation investigating TNF-α, IFN-γ, IL-2, and IL-1β in mice after sildenafil (82).

As from studies in human males and females, sildenafil influences the expression of Treg transcription factor Foxp3, involved in T effector cell deactivation, attenuates NK activity and TNFα level (83, 84), Albeit some controversies on reported data, there is evidence on sildenafil-induced reduction of local inflammatory mediators and receptors, either local or circulating, such as IL-1β, TNFα, monocyte chemoattractant protein 1(MCP-1) and its receptor CCR2, documented in different animal model of diseases (82).

It is worthwhile to mention that overproduction of reactive oxygen species (ROS), characterized by an imbalance in the synthesis and removal of ROS, commonly detected in cardiovascular disease, can be accompanied by reduced endothelium-derived NO responses (85), and impairment of soluble GC function, which might contribute to the increased tone seen in pathophysiological conditions associated with chronic overproduction of ROS. A direct link between enhanced ROS and decreased sGC expression was established by Gerassimou and colleagues (86), showing that exposure of smooth muscle cells to exogenously applied ROS-generating agents caused a decrease in α1 and β1 sGC subunit levels, thus attenuating cGMP formation. Since cGMP is engaged in the regulation of smooth muscle cell proliferation as well as vessel tone, the inhibition of sGC expression by ROS in pathophysiological states would be expected to modulate vascular remodeling and tone. Reduction in the availability and function of NO contributes to vasoconstriction, inflammation and vascular remodeling. Remarkably, whilst playing a key role in the pathogenesis of cardiovascular disease, ROS dysregulation can also limit the clinical efficacy of current therapies (87).

### 3.2 PDE5i and inflammatory mediators: an example from CXC chemokines for cardiac purposes

The effect of PDE5i onto immune status largely accounts for the indication “back” for cardiac purposes, as previously stated in this review.

Indeed, the role of fine-tuned immune/inflammatory processes, especially driven by Th1 type dominance, is undeniably recognized among the mechanism associated with or preceding cardiac disorders. We have reported on the importance of early changes in circulating and local concentrations of some biomediators, such as CXC-motif chemokines, early involved in the shift towards cardiac diseases, before clinical sign manifestations. Hence, it is of pivotal importance to control as soon as possible their biological effects to control/limit disease initiation.

This is the case of the chemokines CXCL10 and CXCL8, classified as ERL- and ERL+, based on absence or presence of Glut-Leu-Arg motif (88), both molecules with multifaceted functions in disease development, widely engaged in cardiac disease initiation and progression (89).

Interestingly, CXCL10 and CXCL8 significantly increased in sera of diabetic subjects at the onset of cardiomyopathy and are targeted by sildenafil (59, 90). Besides the decrease in systemic level of these CXC molecules, sildenafil can inhibit their release from human endothelial cells and cardiomyocytes, suggesting an anti-inflammatory action at local level (90). This effect is, indeed, quite remarkable since the local cellular components are dynamic units able to interact with and potentiate the signaling inflammatory network, when challenged by Th 1-driven microenvironment, i.e., due to sugar or lipid excess (91, 92). Cardiomyocytes or endothelial cells can contribute to the immune/inflammatory dialogue between local and systemic counterparts producing, among other proinflammatory mediators, CXCL10 and CXCL8, both virtually absent in non-inflammatory conditions; this effect recalls and amplifies local infiltration by immunocytes, triggering an aberrant cell remodeling and establishing a vicious loop (91). Interestingly, sildenafil-induced inhibition of IL-8 from human endothelial cells associates with cGMP increased concentration (90). Higher CXCL10 and CXCL8 levels detected in patients with different cardiac diseases vs. healthy subjects likely reflect early inflammatory processes, differently from classical biomarkers, which reveal already established damages (91). Interestingly, three-month treatment with sildenafil (100 mg/day) decreased CXCL10 and CXCL8 in subjects with diabetes at onset of cardiomyopathy, ameliorated metabolic indices like post-prandial glycemia, hemoglobin A1c, lipidemic profile, whereas failed to modify cardiac standard markers, such as mass and volume index, ejection fraction, blood pressure (93, 94). The latter result is likely due to the well-preserved left ventricular function of the subjects enrolled at the onset of cardiomyopathy, with no sign ischemia; at the same time, this observation suggests the importance of PDE5 inhibition at the onset of cardiomyopathy, to decline early inflammatory biomediators. Decreasing chemokines like CXCL10, known trigger the first auto- or alloimmune response, and CXCL8, present since early stages of inflammation and remaining active for long time (95), before clinical manifestations - i.e., at stages A or B, according to American Heart Association/American College of Cardiology (AHA/ACC) classification (https://www.heart.org/en/health-topics/heart-failure/what-is-heart-failure/classes-of-heart-failure) - might be of clinically relevant impact, as it could attenuate downstream inflammatory cascade and progression to HF. In line with this observation, higher level of inflammatory cytokines associates with increased risk of adverse cardiovascular outcome (96, 97), pretransplant higher CXCL10 correlates with higher risk of heart rejection and worse outcome (98), and trials designed to target inflammatory cytokines to improve outcome are reported (99) there are ongoing trials. Recently, integrated multi-omics data show decrease in CXCL10 is a promising drug target, since it accounts for about 70% of the effects mediated by pharmacological IL-6R inhibition in a clinical trial (100).

Indeed, albeit HF pathophysiology exhibits remarkable heterogeneity, anti-cytokine therapy emerges as one of the most promising approaches. Quite intriguingly, the hypothesis of a “proinflammatory cytokine storm” proposed by Seta et al. in 1996 has been strengthened as research on cardiac diseases has progressed, highlighting the importance of inflammation as one of the main therapeutic targets (101).

A similar process involving a chemokine storm is likely to trigger a significant overresponse in autoimmune diseases, such as Systemic Lupus Erythematosus (SLE) (102, 103). Remarkably, the chemokine CXCL10 seems to act as a trigger/enhancer of the immune storm. This effect is not surprising, since CXCL10 is engaged in early processes both in allo- and auto-immune response, as previously addressed (104).

In Systemic Sclerosis (SSc), an autoimmune disorder characterized by important vascular impairments and multiorgan severe failure (105), CXCL10 and CXCL11 can discriminate those subjects developing SSc from an initial “lighter” condition of very early diagnosis of SSc (VEDOSS) (106). Of note, PDE5i, which are recommended as first-line agents for the treatment of secondary PAH, and for the management of refractory Raynaud’s phenomenon (46, 107, 108), could result useful to maintain cardiac and skeletal muscle function, as previously reported (109).

## 4 Role of PDE5 in cancer

Cyclic nucleotides act as second messengers promoting protein kinase activation, which in turn can promote apoptotic signaling. The cGMP and cAMP signaling pathway is aberrantly regulated in several human cancers, displaying different characteristics. Both cAMP and cGMP were shown to display antiproliferative and pro-apoptotic properties (110). In line with these observations, altered expression of PDE isozymes was reported in different cancer specimens and in hematological malignancies (111, 112).

In this paragraph, we will provide a detailed overview of clinical data on PDE5 expression and function in the pathogenesis of human cancer. In addition, we will discuss whether PDE5 inhibitors could be exploited for therapeutic purpose in cancer, evaluating ongoing clinical trials, summarized in **Table 1**.

**Table 1:** Clinical trials for human cancer treatment using PDE5i.

NCT number	Study title	Intervention	Study status	Conditions	Duration	Reference studies
**NCT02466802**	Study of Regorafenib and Sildenafil for Advanced Solid Tumors	Regorafenib, Sildenafil Citrate	COMPLETED	Solid Tumor	2015-2020	(153)
**NCT02106871**	Study of Sildenafil as a Therapy for Fatigue in Pancreatic Cancer	Sildenafil	WITHDRAWN	Pancreatic Cancer/ Cholangiocarcinoma	2014-2017	Withdrawn/ No results posted
**NCT01375699**	Doxorubicin With or Without Sildenafil, With Analysis of Cardiac Markers	Doxorubicin, Sildenafil	COMPLETED	Breast Cancer	2011-2018	(130)
**NCT03785210**	Nivolumab (Anti-PD1), Tadalafil and Oral Vancomycin in People With Refractory Primary Hepatocellular Carcinoma or Liver Dominant Metastatic Cancer From Colorectal or Pancreatic Cancers	Nivolumab, tadalafil, oral vancomycin	COMPLETED	Metastatic pancreatic Cancer, Metastatic colorectal cancer, Hepatocellular carcinoma	2019-2022	Not disclosed
**NCT01290484**	A Study to Evaluate Sildenafil for the Treatment of Lymphatic Malformations	Sildenafil	COMPLETED	Lymphangioma	2010-2015	(154)
**NCT01950923**	Sildenafil Citrate Before Surgery in Improving Kidney Function in Patients With Kidney Cancer	sildenafil citrate, placebo; therapeutic conventional surgery	COMPLETED	Kidney Tumor	2013-2016	No results posted
**NCT02335242**	Sildenafil for the Treatment of Lymphatic Malformations	Sildenafil; Placebo	COMPLETED	Lymphatic Malformations/ Lymphatic Diseases	2015-2021	(154, 155)
**NCT00165295**	Sildenafil Citrate in Waldenstrom’s Macroglobulinemia	Sildenafil citrate (Viagra)	COMPLETED	Waldenstrom’s Macroglobulinemia	2005-2011	No results posted
**NCT01817751**	Sorafenib, Valproic Acid, and Sildenafil in Treating Patients With Recurrent High-Grade Glioma	sorafenib tosylate; valproic acid; sildenafil citrate	ACTIVE	Glioblastoma	2013-2023	(147, 148)
**NCT00752115**	Combination Chemotherapy With Sildenafil Plus Carboplatin and Paclitaxel in Patients With Advanced Non-small Cell Lung Cancer	Sildenafil; placebo; paclitaxel (taxol); carboplatin (palaplatin)	COMPLETED	Non-small Cell Lung Cancer	2007-2010	No results posted
**NCT02279992**	Pilot Study of Vardenafil and Carboplatin in Patients With Gliomas and Brain Metastases	Vardenafil; Carboplatin	TERMINATED	Glioma Brain Neoplasms Brain Metastasis	2012-2016	No results posted
**NCT03259516**	Nivolumab With Chemotherapy in Refractory MDS	Nivolumab; Azacitidine; Fludarabine; Cyclophosphamide; Cytarabine; all trans retinoic acid; Sildenafil; Melphalan	TERMINATED	Myelodysplastic Syndromes	2017-2019	No results posted

### 4.1 Prostate cancer

Several prostate cancer (PCa) cell lines display increased level of PDE5 expression (113), and PDE5 inhibitors can sensitize cancer cells to chemotherapeutic agents, either by reducing extrusion of the chemotherapeutics agents by ABCB1, ABCC1 and ABCG2 transporters (114) or by promoting apoptosis (115). The pathway relies on the upregulation of caspase 3 and 9, downregulation of Bcl-XL and production of reactive oxygen species (ROS) (116). In xenograft mouse models of PCa, sildenafil significantly inhibited tumor growth by inducing apoptosis and increasing the expression of activated caspase 3, while protecting mice against reperfusion injury/ischemia, which is a side effect induced by doxorubicin treatment (116). In addition, in PCa cells, hypoxic conditions can inhibit the NO/cGMP pathway, leading to chemoresistance. Thus, treatment with PDE5 inhibitors sensitizes hypoxic cells to chemotherapeutic agents by restoring the NO/cGMP pathway (117). PDE5 inhibitors can also counteract PCa progression by increasing apoptosis, as shown in combined therapy with cisplatin or doxorubicin (118). However, VCaP cells, which display the TMPRSS2-ERG fusion, display increased risk of PCa progression after treatment with PDE5 inhibitors (119), demonstrating that PCa cells display different properties and behavior in response to PDE5 inhibition.

PCa can evolve into a castration resistance and more aggressive stage, in which androgen deprivation therapy is not efficacious anymore (castration-resistant prostate cancer -CRPC). During this stage, current treatment is represented by vincristine, which is an anti-mitotic agent, which induces cell cycle arrest and caspase-mediated cell death, but as side-effect can favor the occurrence of neuropathies. Combination of vincristine with sildenafil can reduce deleterious side effects of vincristine, by lowering effective concentration of the chemotherapeutic agent (120). Moreover, Sildenafil was shown amplify vincristine effect both *in vitro* (PC-3 and DU-145 cell lines) and *in vivo* (120).

Sildenafil has been extensively used in clinical trials for PCa patients to recover erectile dysfunction following hormone therapy, radiation therapy or prostatectomy. Thus, these trials have not been addressed in the present manuscript.

### 4.2 Breast cancer

Increased PDE5 expression was shown in clinical samples of breast cancer patients, at both RNA and protein level, and was correlated with tumor grade, stage of the disease and lymph node involvement (121, 122). PDE5 was also found differentially expressed in breast cancer subtypes, with higher expression levels in the more aggressive HER2-enriched and triple-negative subtypes and lower levels in the estrogen receptor (ER)-positive Luminal B- and the Luminal A subtypes (123). Remarkably, high PDE5 expression was correlated with worse prognosis in a cohort of 1,988 patients (123).

PDE5 inhibition with sulindac sulfide (SS) was shown to promote cGMP accumulation, activation of PKG which, in turn, caused inhibition of cell growth and apoptosis in breast cancer cell lines (124), representing a viable strategy for breast cancer treatment. Similarly, sildenafil and tadalafil treatment of breast cancer cell lines diminished cell growth and induced apoptosis, at least in part by modulating β-catenin expression transcription (125). Sildenafil treatment was also shown to affect HSP90 expression, thus promoting PKD2 degradation and impairing cancer cell proliferation and viability (126).

PDE5 activity supports stromal fibroblasts, which produce and secrete chemokines like CXCL16, promoting cancer progression. Sildenafil and vardenafil treatment of breast cancer associated fibroblasts reduced CXCL16 chemokine expression, thus inhibiting cancer progression and increasing the efficiency of both chemotherapy and radiotherapy (127). Importantly, sildenafil can act as an aromatase inhibitor, impairing the conversion of androgens into estrogens, thereby inhibiting breast cancer growth (128). By improving blood supply to the tumor vasculature, PDE5 inhibitors can also ameliorate antineoplastic drug administration in the tumor region, thus reducing tumor size in mouse xenograft models (129).

Despite these relevant observations, only one study clinically evaluated the safety and activity of PDE5 inhibitors in breast cancer. Particularly, it was shown that administration of exisulind in combination with capecitabine was well tolerated in a small number of patients with metastatic breast cancer (n = 35), but the synergism between these two drugs at the doses tested was modest. The clinical trial NCT01375699 evaluated the use of sildenafil as a cardioprotective factor after doxorubicin chemotherapy, in breast cancer patients. Although the results are still being processed, this study demonstrated that adding sildenafil to chemotherapy was safe (130) 5these reported findings suggest that PDE5 inhibitors could be used as adjuvants in breast cancer treatment, decreasing cancer aggressiveness and avoiding cardiovascular side effects caused by conventional chemotherapies.

### 4.3 Colorectal cancer

Inflammation can represent a driver of oncogenesis; in fact, patients with inflammatory bowel disease (IBD) display increased risk to develop colorectal cancer (CRC), thus providing rationale for using anti-inflammatory drugs in CRC prevention. Notably, intestinal cGMP signaling regulates epithelial homeostasis and was implicated in the suppression of colitis and colon cancer. In this context, inhibition of PDE5 could be instrumental to counteract CRC. Treatment of mice with sildenafil was sufficient to activate cGMP signaling in the colon mucosa and to protect against azoxymethane/dextran sulfate sodium (AOM/DSS) inflammation-driven colorectal cancer. Particularly, oral administration of sildenafil in mice after treatment with AOM/DSS, reduced polyp formation and inflammation, also counteracting myeloid-cell infiltration (131). These results highlight the potential therapeutic value of PDE5 inhibitors for the prevention of colitis-driven colon cancer. In line with these results, clinical studies confirmed the therapeutic value of PDE inhibitors, such as exisulind, in the prevention of colorectal polyp formation in patients with familial adenomatous polyposis (FAP) (132, 133).

Furthermore, PDE5 inhibitors were shown to inhibit the oncogenic activity of β-catenin either by repressing its transcription or promoting its proteosomal degradation in several colon cancer cells (134–136). Treatment of human CRC cells with sildenafil resulted in the inhibition of cell proliferation, cell cycle arrest and apoptosis; moreover, it showed reduction of tumor growth in CRC xenograft models in nude mice (137).

Combined therapy of tadalafil with nivolumab and vancomycin was evaluated in the clinical trial NCT03785210, demonstrating decreased liver metastasis from primary colorectal and pancreatic cancer (**Table 1**). This result suggests that sildenafil could be used in combination with other drugs in the treatment of CRC.

### 4.4 Lung cancer

Several lung cancer cell lines were shown to overexpress PDE enzymes, resulting in the decrease of cGMP. Hence, treatment with PDE inhibitors should result in increased intracellular cGMP and inhibition of tumor growth (138). This hypothesis was tested in several studies exploring the potential of combined therapies of PDEi with classical cytotoxic agents.

Combined use of platinum-based agents with PDEi displayed higher antiproliferative effect on lung cancer cells in comparison with the platinum monotherapy, which is currently in use as the standard care (139). Similar effects were also observed by combining sildenafil with radiotherapy in the treatment of Lewis lung carcinoma (LLC), where inhibition of PDE activity was instrumental to abolish the irradiation-derived immunosuppression, improving the T cells response. These results suggest that sildenafil could be exploited to delay tumor recurrence after radiotherapy (140).

The clinical trial NCT00752115 is currently evaluating the combined treatment of sildenafil and carboplatin in patients with advanced non-small-cell lung cancer (**Table 1**).

### 4.5 Brain cancer

PDE5 is highly expressed in several brain tumor cell lines, including glioma and glioblastoma. Moreover, it was found upregulated during murine neuroblastoma cell differentiation (141).

Remarkably, modulation of cGMP signaling via PDEi was shown to improve the permeability across the brain blood barrier, thus improving delivery of therapeutic agents. Oral administration of sildenafil and vardenafil was shown to enhance tumor capillary permeability in rat models of gliosarcoma, thus improving the efficacy of the chemotherapeutic treatment (142). Moreover, combined administration of adriamycin and vardenafil in rat model of brain tumors showed longer overall survival than adriamycin alone (142). Similarly, combined therapy of sildenafil and OSU-03012 improved the efficacy of the therapy in glioblastoma cells by decreasing the expression of plasma membrane receptors and drug efflux pumps (143). In hard-to-treat brain metastases from different primary tumors, PDE5 inhibitors effectively modulated the brain blood barrier permeability, enhancing delivery and therapeutic efficacy of monoclonal antibodies in mouse models (144, 145).

However, in glioblastoma multiforme, which is the most aggressive and lethal brain tumor, high levels of the PDE5 protein were associated with decreased aggressiveness, invasive potential, and resistance to radiation (146).

The phase II clinical trial NCT01817751 is evaluating the activity of sildenafil and other drugs (sorafenib and valproic acid) in patients with recurrent high-grade glioma (147, 148), whereas the clinical trial NCT02279992 is evaluating the use of vardenafil to increase systemic concentration of carboplatin in patients with recurrent malignant gliomas or metastatic brain cancer. The NCT01817751 study confirmed the safety and survival benefit of the 3-drug combination sildenafil-sorafenib-valproic acid for the treatment of high-grade glioma (148). The combination of sorafenib and valproic acid was predicated on the basis that sorafenib activity is enhanced by HDAC inhibition (149, 150), whereas the addition of sildenafil was based on its ability to increase peroxynitrate levels and to block ABCB1 and ABCG2 drug-efflux pumps, thus increasing drug delivery to the tumor (114, 151).

PDE5i are being evaluated also in other tumor subtypes, not discussed in this manuscript. These trials were summarized in **Table 1**.

## 5 Concluding remarks

So far, even though the fortune of PDE5i started and continues as drugs for ED treatment, it is undeniable that their potential administration in inflammatory-related diseases amplifies their range of possible applications. Particularly, PDE5i, as anti-inflammatory drugs, potentially have the power to revert destabilized cell homeostasis, that is the turning-point where several diseases start from considering the many favorable effects deriving from PDE5i-dependent cGMP stabilization, the clinical exploitation of PDE5i can go undeniably beyond ED, as we addressed here, particularly for cardiac diseases and cancer. Efforts from the scientific community are to recognize and close the gaps present in knowledge, to address, expand and diversify the potential clinical use of PDE5i.

Indeed, albeit there are promising data on PDE5i administration to reduce adverse cardiac outcome, particularly in comorbidity with diabetes, consistent translation to other cardiac diseases, such as infarction, CHF, arrhythmia, is still lacking. Some reasons for the limitation in PDE5i translatability from bench to bedside may include, i.e., drug dosages or the use of animal models, not always immediately translatable to humans. Despite previous disappointments in clinical translation, targeting inflammation is the most promising and attractive direction in cardiovascular therapeutics. Dissection of the paths and mechanisms underlying aberrant responses as well as a better profiling of patient subpopulations will improve the benefit from PDE5i-targeted inflammation.

Moreover, albeit large-scale studies and trials support the anti-cancer potential of PDE5i, the association between PDE5 inhibition and tumor microenvironment, universally acknowledged to be critical in driving tumor growth/invasion/metastasis, and response to therapies, is still lacking. Furthermore, since PDE5i are not free of side effects, i.e., headaches, flushing, dyspepsia, altered vision and optic neuropathy, back pain and myalgia, priapism, melanoma, blood pressure, rhinitis, prostate cancer, hearing loss (152). Thus, well-performed studies on selected populations are needed to identify the populations with a “safe” profile to undergo PDE5i treatment. In this scenario, further studies aimed to validate PDE5i clinical applications are mandatory to expand our understanding, in a view of precision and interdisciplinary approach.
